# Dasatinib-Induced Gynecomastia: A Case Report

**DOI:** 10.7759/cureus.76121

**Published:** 2024-12-21

**Authors:** Himanshi Kaushik, Kanchan D Devde, Nishtha Manuja, Rahul Arora, Amol Dongre

**Affiliations:** 1 Medical Oncology, Jawaharlal Nehru Medical College, Wardha, IND; 2 Medicine, Jawaharlal Nehru Medical College, Wardha, IND

**Keywords:** blast cell, bone marrow aspiration, chronic myeloid leukemia, testosterone, tyrosine kinase inhibitor

## Abstract

Gynecomastia, the abnormal enlargement of male breast tissue, is a rare side effect associated with dasatinib. This drug is used in the treatment of chronic myeloid leukemia (CML). We present a case of dasatinib-induced gynecomastia in a 52-year-old gentleman with CML who developed bilateral breast enlargement and tenderness after approximately four months of dasatinib treatment. The patient's hormonal profile was within normal limits, including testosterone, estradiol, prolactin, and follicle-stimulating hormone levels. While the exact pathophysiology remains unclear, it is postulated that dasatinib's inhibition of various kinases, including src family kinases and receptor kinases, may contribute to developing gynecomastia. The reported incidence of dasatinib-induced gynecomastia is low, and the onset of symptoms can vary widely. Management strategies for dasatinib-induced gynecomastia are not well-established, but options include androgen support, tamoxifen, or switching to an alternative tyrosine kinase inhibitor. This case report highlights the importance of monitoring patients on dasatinib therapy for developing gynecomastia and other hormonal abnormalities. Clinicians should educate the patients about the possibility of this potential adverse effect, emphasizing the need to report any symptoms indicative of low testosterone syndromes. Further research is warranted to better understand the underlying mechanisms, risk factors, and optimal management strategies for dasatinib-induced gynecomastia.

## Introduction

Chronic myeloid leukemia (CML) is a myeloproliferative neoplasm that accounts for approximately 15% of adult leukemia cases [1]. CML is characterized by the clonal expansion of myeloid cells at varying stages of differentiation, driven by a specific genetic abnormality known as the Philadelphia chromosome. This chromosomal abnormality results from a reciprocal translocation between chromosomes 9 and 22, leading to the formation of the BCR-ABL1 fusion gene. The BCR-ABL1 gene encodes a constitutively active tyrosine kinase, BCR::ABL, which plays a central role in the pathogenesis of CML by promoting uncontrolled cell proliferation and inhibiting apoptosis [2].

Selecting the appropriate tyrosine kinase inhibitor (TKI) therapy is a nuanced decision that involves evaluating various factors such as the patient’s age, comorbidities, tolerance to treatment, and risk score. While TKIs are generally well-tolerated, they are associated with a range of side effects, including gastrointestinal disturbances, edema, musculoskeletal pain, and dermatological reactions [3,4].

Among these, gynecomastia is a rare but notable side effect that has been reported in association with both imatinib and dasatinib therapy [5]. The incidence of dasatinib-induced gynecomastia is noted to be less than 1% [4].

Gynecomastia, defined as the benign proliferation of glandular breast tissue in males, typically results from reduced androgen sensitivity in the breast tissue and increased estrogen production due to elevated androgen precursors. Androgen resistance at the pituitary raises luteinizing hormone and testosterone levels, but the hormonal imbalance still leads to breast growth. Although commonly associated with hormonal therapies, non-hormonal drugs, including TKIs, have also been implicated in its development. Medications can cause gynecomastia through several mechanisms, such as increasing estrogen levels or mimicking estrogen activity, lowering testosterone levels, causing hypogonadism (reduced function of the testes), having anti-androgenic effects (blocking male hormones), or raising prolactin levels [6]. The exact mechanism by which TKIs induce gynecomastia is not fully understood, but it is hypothesized that inhibition of specific kinases involved in steroidogenesis and testicular function may play a role.

In this case report, we present the occurrence of gynecomastia in a 52-year-old male undergoing treatment for CML with dasatinib. This case highlights the importance of recognizing rare but significant adverse effects associated with TKIs, which can impact the patient’s quality of life and influence treatment decisions.

## Case presentation

**Figure 1:**
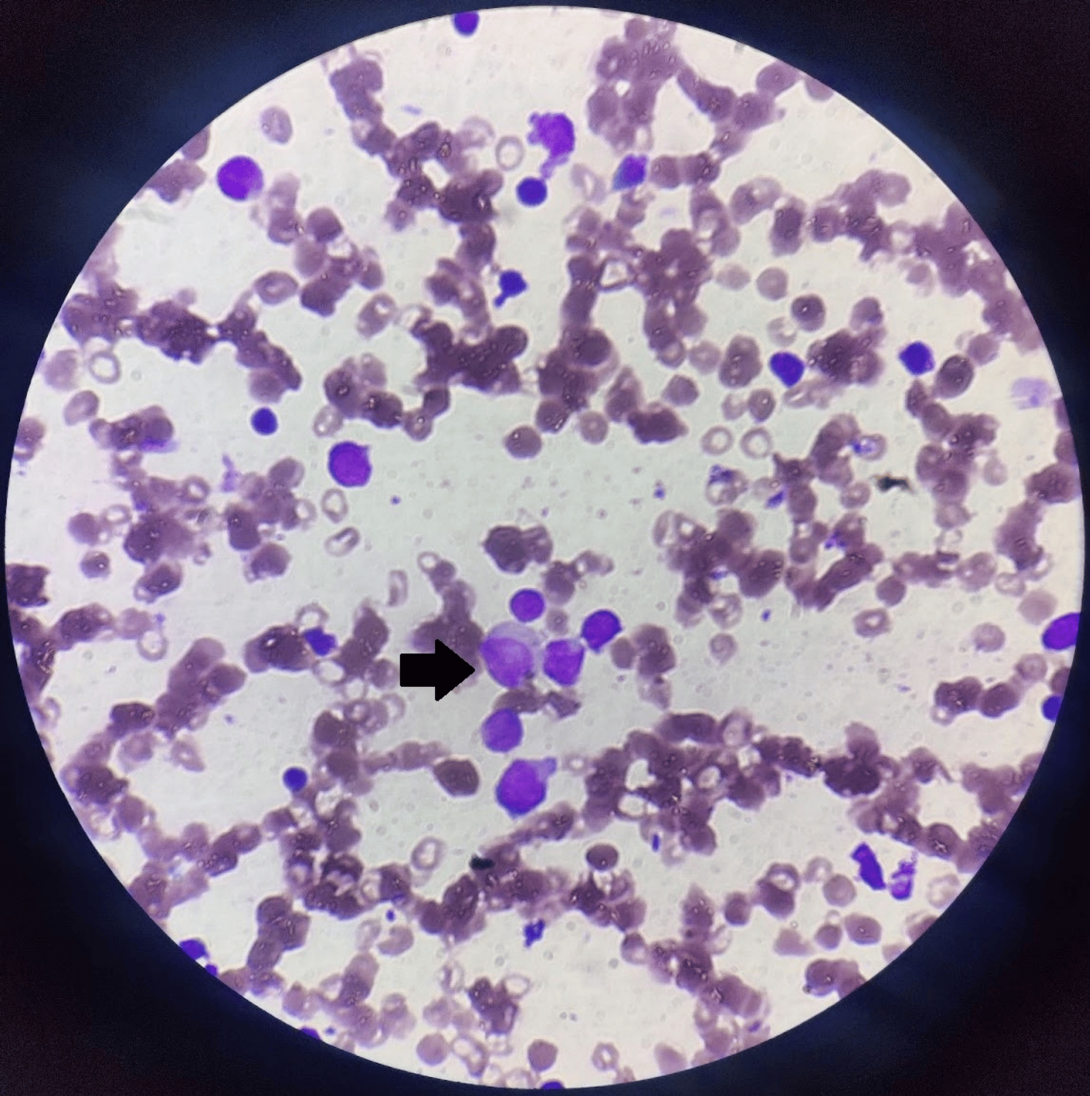
Peripheral blood smear showing blast cells (black arrow) suggestive of chronic myeloid leukemia

**Figure 2:**
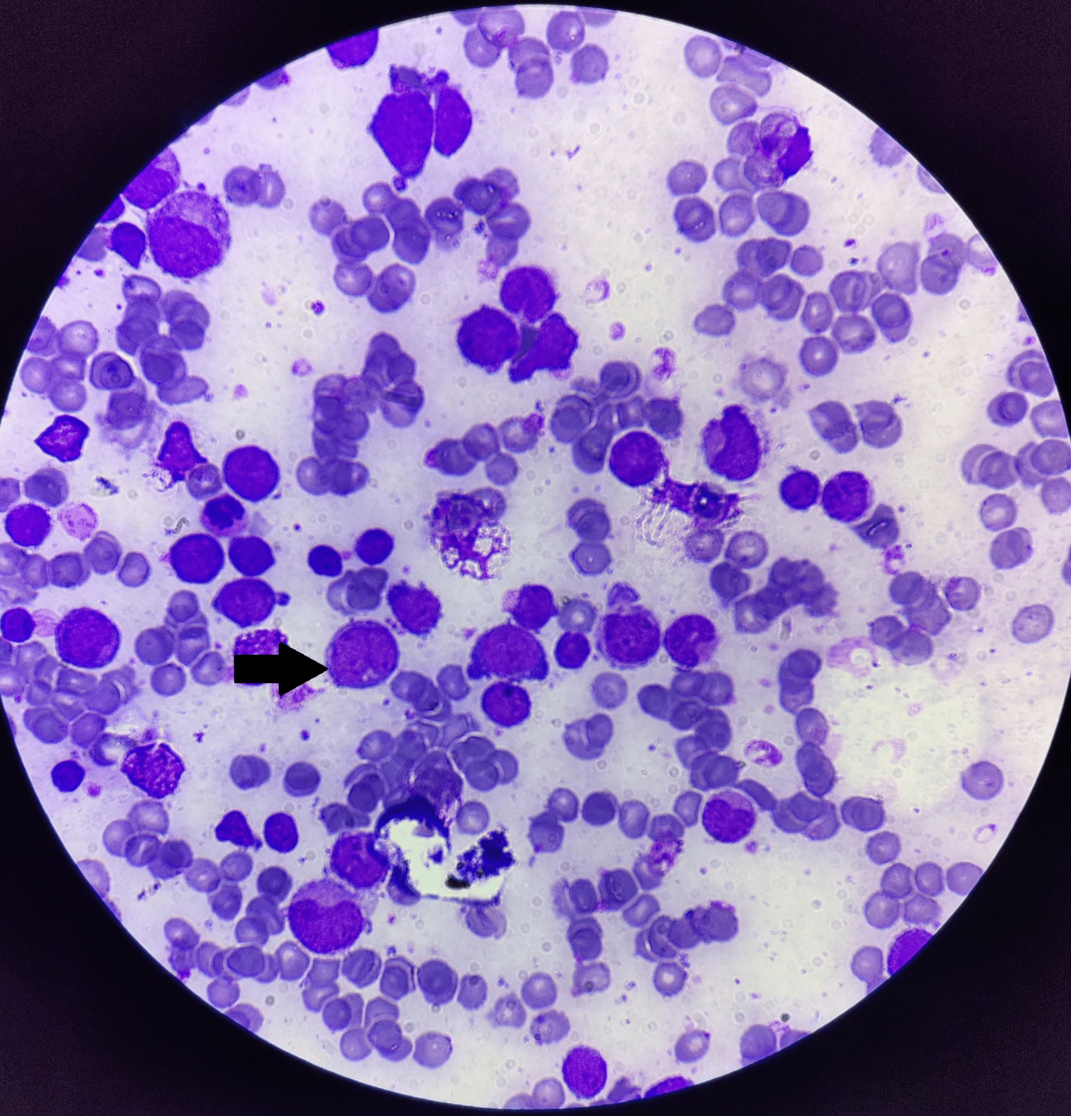
Accelerated phase in bone marrow aspiration (black arrow)

A 52-year-old male presented with a 15-day history of fever, generalized weakness, and easy fatigability. On evaluation, he was found to have anemia, leukopenia, and thrombocytopenia (Table 1) with mild hepatosplenomegaly. Based on the findings from his peripheral smear (Figure 1) and bone marrow aspiration report (Figure 2), he was diagnosed with CML in the accelerated phase, with 19% blast cells in September 2022. Dasatinib 100 mg daily was initiated as first-line therapy.

**Table 1:** Laboratory Investigations

Parameter	Initial Presentation (September 2022)	Follow-Up (After 4 Months)	Reference Range
Hemoglobin (g/dL)	5.8	10.2	13.0-17.0
White Blood Cells (× 10^9^/L)	2.09	4.5	4.0-11.0
Platelets (× 10^3^/µL)	24	150	150-450
Blast Cells (%)	19	2	<5

After about 4 months of treatment, the patient began to experience pain and noticed an increase in the size of both breasts. He reported that bilateral breast enlargement and tenderness had been worsening over the last 10 days. The patient denied any changes in his medication regimen, weight, or exercise routine during this period. He had no prior history of gynecomastia or other breast disorders before starting dasatinib.

**Figure 3:**
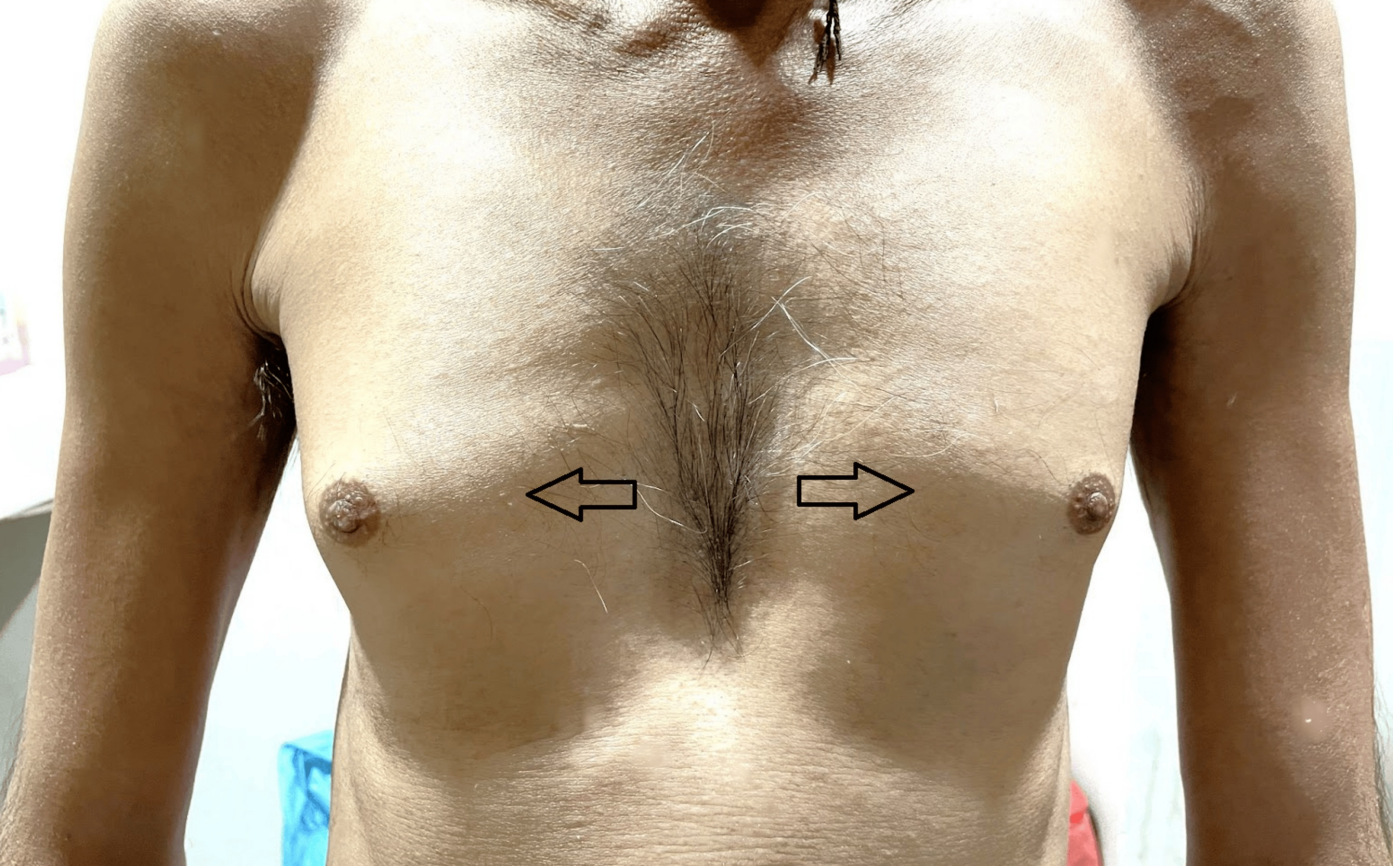
Bilateral gynecomastia (black arrows) in a thin-built male

On physical examination, the patient was noted to have bilateral breast enlargement, with firm and tender glandular tissue palpable beneath the areolae (Figure 3). The right breast seemed to be slightly more enlarged than the left one. No lumps/nodules were palpable in both breasts. There were no signs of inflammation, redness, infection, or trauma. There was no discharge, bleeding from the nipple, or any skin changes. No lymph nodes were palpable in the bilateral axilla. The rest of the physical examination was unremarkable.

Laboratory investigations were performed to evaluate the hormonal status of the patient. His hormone profile was within normal limits (Table 2).

**Table 2:** Hormonal profile FSH, Follicle-stimulating hormone

Hormonal Profile	Values	Normal Values
Testosterone	295 ng/dL	270-1,070 ng/dL
Estradiol	20.598 pg/mL	10-50 pg/mL
Prolactin	12.1 ng/mL	<20 ng/mL
FSH	7.42 mIU/L	1.5-12.4 mIU/L

With the clinical presentation and lab findings, the engorgement of the breast was suggestive of dasatinib-induced gynecomastia. Careful consideration was taken of the risks and benefits while continuing the current TKI compared to switching to an alternative TKI. The patient opted to maintain the treatment with dasatinib, accompanied by careful monitoring. This involved regular physical exams during follow-up appointments and encouraging the patient to report any increases in breast tissue or any painful symptoms. The patient was advised to continue dasatinib therapy, as it was critical for his CML management. At the two-month follow-up visit, there was no increase in the size of both breasts. The patient reported a significant improvement in breast tenderness. A repeat USG showed a decrease in the size of the glandular tissue.

On physical examination, there was a slight reduction in the size of both breasts. Firm and non-tender glandular tissue is palpable beneath the areolae. There was no inflammation, redness, infection, or trauma in the breast area. There were no indications of nipple discharge or changes in the skin. No lymph nodes were palpable in the bilateral axilla. The remaining aspects of the physical examination did not uncover any noteworthy abnormalities. No history of alcohol consumption or any additional medications were reported in conjunction with dasatinib. No other obvious or conceivable causes of gynecomastia were identified. There were no changes observed in the size of gynecomastia even after the continuation of dasatinib, which remained stable and hence continued.

## Discussion

TKIs have completely changed the way that CML is treated, increasing overall survival rates and making the illness much more tolerable for a large number of patients. Similar to imatinib, dasatinib is generally well tolerated and effective, although more research is needed to fully understand its range of mechanisms and adverse effects.

Dasatinib is a second-generation TKI that is frequently used for the management of CML. Dasatinib works by blocking receptor kinases such as c-KIT, PDGFR, DDR 1 and 2, c-FMS, ephrin receptors, BCR-ABL, SRC family kinases (v-src sarcoma viral oncogene homolog), and TEC family kinases (TEC and BTK) [3]. It is also known as a "dual inhibitor." It is known to cause several adverse effects, including fluid retention, myelosuppression, and cardiovascular events [2]. 

Gynecomastia is a rare but known adverse effect of dasatinib treatment, accounting for less than 1%. It is characterized by the enlargement of breast tissue in males, which is usually bilateral and symmetrical. The exact mechanism of dasatinib-induced gynecomastia is not well understood. It is hypothesized that dasatinib may disrupt the estrogen-testosterone balance, leading to increased estrogen levels and subsequent breast tissue proliferation. 

The proposed mechanism of gynecomastia, which is caused by dasatinib and imatinib, involves inhibiting the PDGFR and/or c-KIT. These are expressed in the testes and play a role in the production and secretion of testosterone. PDGFR is crucial for the maturation of the testes and differentiation of Leydig cells, while PDGFR-A is necessary for the recruitment of Leydig cells and spermatogenesis, resulting in a change in the estrogen-to-androgen ratio and a decrease in testosterone levels, which can encourage the development of gynecomastia. Gynecomastia in most patients is associated with an increase in progesterone and 17-hydroxyprogesterone, which may be due to reduced testosterone production and an increase in testosterone precursors [3,4,7]. PDGF signaling pathway helps in the regulation of steroidogenic lineages and molecular pathways involved in fertility and hormone production [8].

Since TKI-induced gynecomastia is uncommon, there is no established standard treatment available. The suggested therapeutic methods include administering androgen support, tamoxifen, or switching to an alternative TKI [9,10]. Clinical improvement has been seen with the use of tamoxifen, but when considering treatment with tamoxifen, it is crucial to take into account relevant drug interactions and potentially severe side effects, such as liver toxicity, suppression of bone marrow function, and thromboembolic events [10-12]. If a medication is suspected to be causing gynecomastia, it is recommended to stop or switch the drug, if possible. Switching to a similar drug that has a lower risk of causing gynecomastia may help prevent it from coming back. Within a month of stopping the medication, the breast tenderness and tissue swelling should start to improve. However, if the gynecomastia has been present for more than a year, it may become difficult to reverse due to the development of scar tissue (fibrosis). In such cases, surgery might be needed to remove the excess tissue [13].

The literature has extensively documented the occurrence of gynecomastia induced by imatinib in patients with gastrointestinal stromal tumors, or CML. The onset of this side effect can vary from as early as one month to as late as 17 months [5,9,12,14,15]. A study conducted by Gambacorti-Passerini et al. observed that only 18% of CML patients were seen with enlargement of breast tissue, i.e., gynecomastia, with the use of imatinib for 6 to 13 months. Additionally, a relationship has been observed between hormonal anomalies and the treatment dose (400 mg versus 600-800 mg). This suggests that a certain dosage of the drug may be required to effectively inhibit the activity of steroidogenic enzymes, potentially indicating a "threshold dose" [5]. Although dasatinib-induced gynecomastia is a rare adverse effect, one possible reason for this could be that the drug was not being routinely used to its full potential when compared to imatinib. A study published in the Journal of Hematology Oncology Pharmacy in February 2022 reported two cases of dasatinib-induced gynecomastia in patients with CML [16]. Patients who have experienced gynecomastia as a side effect of dasatinib or imatinib have also reported experiencing diminished libido, impotence, and hydrocele, which are symptoms associated with low testosterone levels. Decreased muscle mass, a higher incidence of metabolic syndrome, and decreased bone mineral density have all been related to low testosterone. Hot flashes, sadness, and decreased libido are physical signs of low testosterone that can significantly lower a patient's quality of life. In clinical practice, healthcare providers should actively discuss these psychosocial issues with patients and offer psychological support or counseling as needed. Addressing the psychological burden of gynecomastia early in treatment could help mitigate long-term emotional consequences. The study emphasized the importance of monitoring for gynecomastia and other hormonal abnormalities in patients treated with dasatinib. Low testosterone levels are seen with imatinib and dasatinib medication; therefore, it is advisable to check the patient's baseline testosterone levels before initiating dasatinib (or imatinib) therapy. The signs of low testosterone, including impotence, decreased libido, and the onset of hydrocele, should be discussed with patients.

## Conclusions

Gynecomastia induced by imatinib is extensively documented in the literature, but the case reported by G. Hobbs is the only documented evidence of dasatinib-induced gynecomastia, as per our knowledge. In conclusion, dasatinib-induced gynecomastia is a rare but potentially distressing adverse effect of dasatinib therapy. It is important for clinicians to be aware of this complication and to monitor patients on dasatinib therapy for breast enlargement or tenderness. Additional studies are required to establish the appropriate method for monitoring testosterone levels in patients undergoing TKI therapy. Patients should be aware of the possibility of the development of gynecomastia as an adverse effect of TKI treatment, as well as the significance of reporting any symptoms that may indicate low testosterone syndromes.
